# Towards a more inclusive child and adolescent mental health research: Bridging gaps through neuro‐affirmative, transdiagnostic, and participatory frameworks

**DOI:** 10.1002/jcv2.70015

**Published:** 2025-05-16

**Authors:** Alessio Bellato, Asilay Seker

**Affiliations:** ^1^ School of Psychology University of Southampton Southampton UK; ^2^ Centre for Innovation in Mental Health University of Southampton Southampton UK; ^3^ Institute for Life Sciences University of Southampton Southampton UK; ^4^ School of Psychology University of Nottingham Semenyih Malaysia; ^5^ Mind and Neurodevelopment (MiND) Research Group University of Nottingham Semenyih Malaysia; ^6^ Institute of Psychiatry Psychology & Neuroscience King's College London London UK

**Keywords:** ADHD, adolescence, autism, child psychiatry, mental health, neurodiversity, participation, participatory, research, transdiagnostic

## Abstract

The field of child and adolescent mental health research is currently undergoing important shifts. In line with its mission to support accessible child mental health science for all, JCPP Advances has included 12 studies in its June 2025 issue, eight of which are presented in this editorial. These articles reflect how recent changes are influencing research in the field. These include the adoption of transdiagnostic frameworks to better understand shared mechanisms across diagnostic categories, and the growing use of participatory research to involve children, young people, and families in the design of assessments and interventions. The highlighted papers examine emotion regulation in autism, longitudinal pathways to psychopathology, the role of family dynamics and prosocial behaviours, and the development of accessible, inclusive tools and interventions. Together, they showcase how the field is evolving to become more developmentally informed, inclusive, and responsive to the real‐world needs of young people and their support networks.

**Key points**

- Traditional child and adolescent mental health research has largely focused on categorical diagnoses, often overlooking overlapping symptoms and real‐world applicability.
- The June 2025 issue of *JCPP Advances* highlights a shift toward transdiagnostic approaches, and a growing emphasis on involving children, young people, and families in research to enhance relevance and impact.
- Featured studies explore topics like emotion regulation in autism, long‐term pathways to psychopathology, family and prosocial dynamics, and the design of inclusive interventions.
- These developments reflect a more inclusive, affirmative, and real‐world responsive approach to child and adolescent mental health research.

The field of child and adolescent mental health research is undergoing important changes – some of which have been described as “paradigm‐flipping” (Sonuga‐Barke, 2023). These shifts are clearly visible in many recent contributions to JCPP Advances, including the papers published in the June 2025 issue, which we are pleased to introduce in this editorial.

We are witnessing (and will likely continue to witness for years to come) significant changes in how child and adolescent mental health research is designed, funded, and carried out. A growing number of researchers are moving toward transdiagnostic frameworks, such as the Research Domain Criteria (RDoC), to study the shared and overlapping mechanisms that underlie the difficulties experienced by children and young people across different diagnostic groups. Focusing on transdiagnostic processes offers a promising alternative to rigid, diagnosis‐specific models of intervention that often target only a narrow set of symptoms while neglecting co‐occurring behavioural and emotional patterns. Another major shift involves the increasing use of participatory research approaches (MacLeod et al., 2024), in which individuals with lived experience are included in the co‐design of assessment tools and interventions (Yau et al., 2024). This collaborative model helps place young people and families back at the centre of the research process, empowering them to make meaningful contributions to studies that aim to better understand and address the very challenges they live with.

The June 2025 issue of JCPP Advances brings together a high‐quality collection of open‐access papers that reflect these priorities. These articles explore how to improve our understanding of neurodivergence and developmental pathways toward psychopathology, and ways to design evidence‐based, inclusive, and accessible tools and interventions to support children and young people facing mental health difficulties.

Of particular relevance is the clinical review by Greaves (2024), who introduced the Neural‐Preferencing Locus of Control (NP‐LOC), a new conceptual framework for understanding emotion regulation differences in autism. Emotion regulation, as described in the clinical review, is a key target of many interventions designed to improve well‐being and daily functioning in autistic individuals, including those with demand‐avoidant presentations. We already know that emotion regulation difficulties are a core concern across neurodevelopmental conditions, and it is becoming increasingly common for clinicians to hear how ‘demand avoidance’ can be a major disruptor in the daily lives of autistic children and their families. Therefore, this clinical review comes highly relevant and timely across the field. The model by Greaves (2024) proposes that autistic children may develop a reduced repertoire of adaptive emotion regulation strategies because they tend to rely less on social support and more on fixed routines and rituals from an early age. This, combined with an external locus of control, may contribute to heightened risk for conditions such as depression and anxiety. The paper's biopsychosocial perspective on emotion regulation in autism is an important contribution to a growing body of work aiming to map developmental pathways underlying transdiagnostic vulnerabilities such as emotional dysregulation.

Other articles in the issue focused on longitudinal approaches to understand developmental trajectories. In a large UK sample of over 7000 children, Griffiths et al. (2024) tested whether teacher‐reported language difficulties during the first year of primary school strongly predict later academic achievement and Special Educational Needs (SEN) provision. Teacher ratings of language difficulties at age 5‐6 were linked with data on SEN provision until age 12–13 and scores on several assessments at ages 5–6, 6–7 and 10–11 years from the UK National Pupil Database. The authors found that early language difficulties strongly predicted later academic outcomes, including reading, writing, and mathematics. Close co‐operation with teachers is essential to plan tailored SEN support for children with additional needs and help them thrive. Therefore, Griffith et al.’s work is important for highlighting issues with maintaining SEN support during the transition from primary to secondary school, the value of teacher observations for early identification of academic and behavioural problems in children, and the importance of early support for young children experiencing language difficulties at primary school entry.

The Griffiths et al. (2024) study links very well with Sideropoulos et al.’s (2024) examining the educational transition of children with special needs from a parental perspective. Using a sample of 61 children from the UK (autistic children and those with Down syndrome or Williams syndrome), the authors examined changes in anxiety and adjustment before and after the primary‐to‐secondary school transition, particularly focusing on underlying factors and parental perspectives. They used a parent‐survey and online interviews with the children during the last year of primary school and then again during the first year of secondary school. Although overall anxiety levels (as reported by parents) remained stable, both parents and children expressed significant concerns about bullying and adapting to new environments. These findings align with those of Griffiths et al. in emphasizing the social‐emotional vulnerability associated with school transitions and the need for proactive, individualised support.

Two studies used data from the Adolescent Brain Cognitive Development (ABCD) study to examine predictors of mental health outcomes across development. Reimann et al. (2024) examined bidirectional associations between prosocial behaviour and psychopathology, while Xu et al. (2024) tested the performance of the IDEA‐RS (Identifying Depression Early in Adolescence Risk Score) in predicting depression in 9‐11‐years‐olds. From the analysis of more than 9000 participants, associations between prosocial behaviour and lower general psychopathology and fewer conduct problems (Griffiths et al., 2024), and associations between high family conflict and poor relationships, and depression (Xu et al., 2024), were found. Together, these studies highlight the importance of family context and social functioning as predictive factors and suggest that enhancing prosocial skills and addressing family dynamics could help mitigate future mental health challenges.

A novel assessment tool was presented by Morey et al. (2024), who described the validation of the CompACT‐Y, a youth version of a widely used measure of psychological flexibility in adults. Psychological inflexibility (the tendency to avoid or suppress difficult thoughts, feelings, and situations) is a known transdiagnostic factor associated with anxiety, depression, and emotional dysregulation. In this study, the authors aimed to validate the CompACT‐Y in a sample of more than 300 adolescents between 13 and 18 years old from the UK. The results of this study support the use of this tool for measuring psychological flexibility (and inflexibility), paving the way for further validation in younger and more diverse populations.

Developing evidence‐based, clinically valid and accessible instrument capturing both condition‐specific and more general factors associated with mental health challenges, is indeed a core objective of our field, as highlighted in the paper by Reardon et al. (2024). The authors developed a practical assessment tool to identify primary school children (8–11 years old) with anxiety disorders. An important finding of this study is that parent‐report questionnaires consisting of between two and nine items could identify children with and without anxiety disorders with an acceptable level of accuracy. Secondly, the study highlighted the importance of considering parent‐reports not only focused on anxiety symptoms but also focused on the impact of such symptoms, since this could be helpful for early identification of children experiencing or more likely to experience anxiety in the future.

Waiting times for neurodevelopmental CAMHS remain high (Maciver et al., 2025); therefore, evidence‐based approaches that are both accessible and acceptable to young people and their families are needed now more than ever. Cullingham et al. (2024) provide an excellent example of how co‐design and digital technology can improve clinical practice. The authors presented a model for co‐designing an intervention for autistic children, adapted from the Online Support and Intervention for child anxiety (OSI), a parent‐led programme targeting anxiety (the most commonly occurring mental health challenge for autistic people). OSI is an 8‐session interactive web‐based intervention supporting parents to help their children overcome problems with anxiety through simple interactive and multi‐modal content based on cognitive behavioural principles, with parents having the opportunity of individual weekly consultations with a therapist to individualise the content. The co‐design process was founded on an iterative feedback process between the research team and the Expert Reference Group (ERG), consisting of four parents of autistic children with anxiety problems, an autistic young person with experience of anxiety problems, and two non‐autistic clinicians with experience supporting autistic children with mental health difficulties. Some of the ERG members are also co‐authors of the paper.

The steps followed for the adaptation of the OSI are detailed in the paper (Cullingham et al., 2024) and consisted of an initial evidence synthesis study (literature review) and further evidence gathering, discussion of evidence with the ERG, a qualitative study (semi‐structured interviews with parents of autistic children with anxiety problems and autistic children with anxiety problems), followed by preparation of intervention content and finalisation of the intervention protocol after a focus group with the ERG. This model offers a valuable blueprint for creating neuro‐affirmative, family‐centred interventions grounded in lived experience.

## CONCLUSION

The articles included in the June 2025 issue of JCPP Advances reflect a field in motion; embracing novel transdiagnostic frameworks and approaches and expanding the definition of evidence‐based practices to include the voices of children and young people with lived experience. The eight studies discussed in this editorial further demonstrate how such changes shall continue to inspire us and think about how we define mental health challenges and conditions, which tools and instruments we use to identify such challenges and problems, and what are the most inclusive, accessible, and efficacious strategies to support mental health in children and young people.

As researchers and clinicians, our goal should be to translate our expertise into practices that are inclusive, accessible, and empowering for all children, young people, and their families – benefiting individuals and society as a whole. It is exciting to be part of the move towards this goal of more inclusive science and practice, and even more exciting to see where it will lead.

## AUTHOR CONTRIBUTIONS

**Alessio Bellato**: Conceptualization; writing—original draft; writing—review and editing. **Asilay Seker**: Writing—review and editing.

## CONFLICT OF INTEREST STATEMENT

Dr. Alessio Bellato declares honoraria as Joint Editor of *JCPP Advances* and travel reimbursements from the International Brain Research Organization (IBRO). Dr. Asilay Seker has received funding from NIHR and Wellcome Trust.

## ETHICAL CONSIDERATIONS

Not applicable.

## Data Availability

Data sharing not applicable to this article as no datasets were generated or analysed.
